# Photodynamic Therapy of Novel Photosensitizer Ameliorates TNBS-Induced Ulcerative Colitis *via* Inhibition of AOC_1_

**DOI:** 10.3389/fphar.2021.746725

**Published:** 2021-10-21

**Authors:** Yumei Rong, Ge Hong, Na Zhu, Yang Liu, Yong Jiang, Tianjun Liu

**Affiliations:** ^1^ Tianjin Key Laboratory of Biomedical Material, Institute of Biomedical Engineering, Chinese Academy of Medical Sciences and Peking Union Medical College, Tianjin, China; ^2^ Department of Gastroenterology, The Second Hospital of Tianjin Medical University, Tianjin, China

**Keywords:** ulcerative colitis, gut microbiota, photodynamic therapy, novel photosensitizer, AOC1

## Abstract

Ulcerative colitis (UC), a chronic, nonspecific inflammatory bowel disease characterized by continuous and diffuse inflammatory changes in the colonic mucosa, requires novel treatment method. Photodynamic therapy (PDT), as a promising physico-chemical treatment method, were used to treat UC rats’ model with novel photosensitizer LD_4_ in this paper, the treatment effect and mechanism was investigated. LD_4_-PDT could improve the survival rate of 2,4,6-trinitrobenzene sulfonic acid (TNBS)-induced UC model rats, decrease expression of interleukin (IL)-6, IL-1, tumor necrosis factor (TNF)-*α*, malondialdehyde (MDA), myeloperoxidase (MPO) and increase the expression of glutathione (GSH) and superoxide oxidase (SOD), while protecting the integrity of the intestinal epithelium. LD_4_-PDT treatment could rebuild the intestinal microflora composition and reprogram the colonic protein profiles in TNBS-induced rats to almost the normal state. Proteomics analysis based upon TNBS-induced UC model rats revealed that Amine oxidase copper-containing 1 (AOC_1_) was a potential target of LD_4_-PDT. Novel photosensitizer agent LD_4_-PDT represents an efficient treatment method for UC, and AOC_1_ may be a promising target.

## Introduction

Ulcerative colitis (UC) is a chronic, nonspecific inflammatory disease of unknown etiology (Shivaji et al., 2020). The lesions are mainly located in the large intestine, mostly in the rectum and sigmoid colon, limited to the colonic mucosa and submucosa, showing continuous non-segmental distribution. The main acute clinical symptoms of UC are recurrent abdominal pain, diarrhea, and hematochezia. UC characterized by a wide range of lesions, complex pathogenesis, frequent recurrent attacks, and easy carcinogenesis, has been included in the list of modern refractory diseases by the World Health Organization (WHO). Its morbidity is related to infection, autoimmunity, heredity, and environment (Costello et al., 2019). With the development of economy and westernization of living habits and dietary composition, the incidence of UC in Asian countries is also increasing annually (Chow et al., 2009; Ng et al., 2017). Finding an effective treatment method of UC, to restore the patients’ physical and mental health and relieve the heavy economic burden brought by the disease to the patients’ families and society in general, has become a key scientific problem in clinical practice.

Currently, the clinical treatment of UC mainly focuses on anti-inflammatory, immune-regulatory or surgical treatments (Qiu et al., 2018; Ward et al., 2018), and pharmaceutical intervention has been the most adopted approach. Conventional therapeutic drugs mainly included amino salicylic acids, glucocorticoids, immunosuppressants, antibiotics and microecological agents. These drugs can only temporarily control and relieve symptoms, but cannot fundamentally cure the disease, and are accompanied by defects such as substantial toxicity and side effects, poor maintenance effect and frequent disease recurrence. Surgical treatment is mainly performed on patients with massive bleeding, intestinal perforation, canceration and toxic intestinal dilatation, and generally carries disadvantages such as large trauma, many complications, slow postoperative recovery and frequent disease recurrence (Hindryckx et al., 2015; Hirono et al., 2018; Ward et al., 2018). Therefore, development of an alternative therapy with good curative effect and fewer adverse reactions become a highly desire in UC research.

With the development of pharmaceutical biotechnology, many novel therapeutic methods have emerged, including biological targeted therapy, traditional Chinese medicine, stem cell therapy and photodynamic therapy (PDT) (Sands et al., 2019). Among them, PDT is a new technology being developed internationally. It can selectively act on target tissues and produce a photodynamic response *via* a photosensitizer (Mallidi et al., 2015). PDT has the advantages of rapid onset, strong targeting, low toxicity and side effects, and repeatable treatment. Recently, its clinical indications have been extended from malignant tumors to increasing numbers of benign diseases. The rapid development of endoscopy and fiber optics technology makes it possible to use PDT to treat gastrointestinal diseases and provides a new direction for the clinical treatment of UC. However, few reports regarding PDT treatment of UC (or inflammatory bowel disease). Favre and colleagues (Favre et al., 2011), using 5-aminolevulinic acid (5-ALA) as the photosensitizer to treat mice bearing Crohn’s disease, found that low dose PDT can down-regulate the expression of proinflammatory cytokines and induction of T-cell apoptosis to improve T-cell-mediated colitis with no significant side effects. Reinhard et al. (2015) treated dextran sulfate sodium (DSS)-induced inflammatory bowel disease in mice with the photosensitizer temoporfin and found that PDT could effectively reduce the symptoms of colitis and prevent intestinal cancer.

Despite their potential, photosensitizers were crucial in PDT. However 5-ALA itself has no photosensitive activity; instead it is transformed to protoporphyrin Ⅸ upon activation by ALA dehydrase, the concentration remains low, with uneven distribution and low efficiency of photodynamic reaction (Maisch et al., 2011). The poor solubility of temoporfin in water tends to cause adverse reactions such as neuralgia and suppression of the central nervous system (Senge, 2012). Therefore, it is necessary to develop a better photosensitizer for the treatment of UC with good solubility in water, high bioavailability, high photo response efficiency and fewer adverse reactions.

We designed and synthesized a series of alkaline amino acid modified amino tetraphenyl porphyrin compounds, all of which showed good physical and chemical properties (Meng et al., 2015). One of the compounds, 5,10,15,20-tetra{4-[(S)-2,6-diamino-hexamide] phenyl} porphyrin (LD_4_), with good water solubility, low toxicity and targeting characteristics, showed unique promotion of wound healing and immune regulation, as well inhibition of microbial pathogens in treatment of traumatic infection (Xu et al., 2016; Zhao et al., 2021). On the basis of these results, we sought to determine whether this photosensitizer could regulate the microflora and treat UC.

Amine oxidase copper-containing 1 (*AOC*_*1*_), an amine oxidase containing copper, catalyzes the degradation of compounds such as propylamine and spermine. Some reports have indicated that *AOC*_*1*_ was involved in allergy and immune responses, cell proliferation, tissue differentiation and cell apoptosis (Strolin Benedetti et al., 2007; Shepard and Dooley, 2015; Vakal et al., 2020). Although its mechanism of action *in vivo* is not fully understood, its role in immune cell transport makes it a target for autoimmune and inflammatory diseases (Peet et al., 2011). *AOC*_*1*_ is mainly distributed in the intestines and kidneys. Clinically, plasma *AOC*_*1*_ activity is used to diagnose intestinal integrity (DiSilvestro et al., 1997). Knockdown of *AOC*_*1*_ could inhibit the activation of protein kinase B (*AKT*) and the epithelial-mesenchymal transition (EMT) process (Xu et al., 2020). The aim of this study was to investigate the photodynamic therapeutic efficacy and molecular mechanism of LD_4_ in models of UC.

## Materials and Methods

### LD_4_ Synthesis and Characterization

Synthesis and characterization of LD_4_ was previously reported by our laboratory (Meng et al., 2015). The structure of LD_4_ is shown in Supplementary Figure S1A. The sample was excited by a 650 nm semiconductor laser (WSLS-650-500m-200M-H4; Wave spectrum Laser; China) through a columnar fiber. The energy density of the spot was measured using a light power meter (LM1; Carl Zeiss).

### Experimental Animals

Male Sprague Dawley rats aged 6–8 weeks were purchased from HFK Biosciences Experimental Animals Company (SCXK 2019-0008, China) and raised in specific pathogen-free conditions. All animal experiments procedures were experimented according to the National Institutes of Health Guide for Care and Use of Laboratory Animals, and the protocol was approved by the Laboratory Animal Management Committee/Laboratory Animal Welfare Ethics Committee, Institute of Radiation Medicine, Chinese Academy of Medical Sciences (Approval No. IRM-DWLL-2017092). The ambient temperature was 22 ± 2°C, with relative air humidity of 40–70%, and food and sterile water were fed according to the experimental requirements.

### Induction of Colitis

Rats were fasted for 24 h then anesthetized with 10% chloral hydrate before colitis induction. The UC model was induced using the method described by Morris et al. (1989), Wirtz et al. (2007). Briefly, 30 mg of TNBS (A28757; Innochem) in 0.25 ml of 50% ethanol was injected into rat colon through a 3 mm diameter polyethylene rubber catheter (inserted 8 cm into the proximal anal rectum). Rats were then maintained in a head low, tail high position for 1 min. Occult blood test paper was used to detect feces every day.

### LD4-Photodynamic Therapy Treatment

The rats were randomly divided into six groups (10 rats per group) and treated as follows: 1, control group (normal saline); 2, TNBS group (TNBS enema); 3, LD_4_-PDTL group [TNBS and low-dose LD_4_ (60 μg/kg), both *via* enema]; 4, LD_4_-PDTM group [TNBS and medium-dose LD_4_ (120 μg/kg), both *via* enema]; 5, LD_4_-PDTH group [TNBS and high-dose LD_4_ (240 μg/kg), both *via* enema]; 6, SASP group [TNBS enema and positive control drug, SASP (500 mg/kg), gavage]. With the day of TNBS enema injection as day 0, treatment was initiated at day 7. LD_4_ was administered *via* enema every other day, while SASP was administered *via* gavage at the same time points, for a total of four treatments. Thirty minutes after each treatment, the colon was irradiated with an intensive 650 nm PDT system at an energy density of 25 J/cm^2^, excluding the SASP group, which was not irradiated. Body weight and food intake were daily measured. After all treatments were completed, fecal samples from each group were collected and stored at −80°C for further analysis of 16S rRNA. After 24 h of fasting, all rats were sacrificed and whole colons and blood were collected. The colon tissue was weighed, dissected and stored at −80°C. Parts of the tissue were also fixed with 4% paraformaldehyde. All rats in the experiment were treated according to guidelines set out in the National Institutes of Health’s Laboratory Animal Care and Use Guidelines.

### FITC-Dextran Fluorescence Intensity Test

Intestinal permeability can be semi-quantitatively measured by detecting fluorescence intensity in serum using fluorescent tracers. Two hundred micrograms of FITC-dextran (FD40S; Sigma-Aldrich) powder were weighed and dissolved in 5 ml rat serum, diluted by doubling dilution method for 10 dilutions, and then fluorescence intensity was detected with a Varioskan Flash 3001 enzyme plate analyzer (Thermo Fisher, Waltham, MA) to obtain a standard curve. The standard curve were shown in Supplementary Figure S1C. Following LD_4_-PDT treatment, six rats from each group were fasted for 4 h before intragastric administration of FITC-dextran at a dose of 0.6 mg/g. Blood samples were collected before the animals were sacrificed and serum without hemolysis was collected. Sera were added to a 96-well plate, at 100 μl per well. Fluorescence intensity (Ex/Em: 488/520 nm) was measured with a Varioskan Flash 3001 enzyme plate analyzer. The FITC-dextran content in the rat sera was then calculated from the established standard curve.

### Biochemical Analysis

Cytokines was correlated tightly with the occurrence of the infection, interleukin-6 (*IL-6*), tumor necrosis factor-*α* (*TNF-α*) and interleukin-1 (*IL-1*), while myeloperoxidase (*MPO*), malondialdehyde (*MDA*), glutathione (*GSH*) and superoxide dismutase (*SOD*) were correlated with oxidative stress reaction, which was often occurred in UC. So here we measured this cytokinesis to evaluate the efficacy of the treatment. *IL-6* (SEA079Ra; Cloud-Clone Corp) and *TNF-α* (SEA133Ra; Cloud-Clone Corp) were quantified using commercially available ELISA kits. Standard curves are shown in Supplementary Figure S1D,E. *MPO* (A044-1-1; Nanjing Jiancheng Bioengineering Institute), *GSH* (A006-1-1; Nanjing Jiancheng Bioengineering Institute), *MDA* (A003-1-2; Nanjing Jiancheng Bioengineering Institute) and *T-SOD* (A001-1-2; Nanjing Jiancheng Bioengineering Institute levels in sera were determined by ELISA assay kits according to the manufacturer’s instructions.

### Histopathological Analysis

Harvested colon tissue was dehydrated, embedded and sliced for histopathological analysis. Hematoxylin and eosin staining was performed as previously described (Zhang et al., 2020), and the extent of inflammation was scored according to the literature (Rong et al., 2018).

### Bacterial Diversity Analysis

Fresh, uncontaminated feces were collected from six rats in different cages and stored at −80°C until use. DNA was extracted from feces and measured by Qubit Fluorometer. The mass concentration of the DNA library was greater than 1.0 ng/μl, which was of sufficient quality for use in subsequent experiments. Illumina MiSeq technology was used to amplify and sequence the V3–V4 region of the bacterial 16S rRNA gene. The bacterial 16S ribosomal (r) RNA forward primer sequence was 5′-CCTACGGGNGGCWGCAG-3′ and the reverse primer sequence was 5′-GACTACHVGGGTATCTAATCC-3′. USEARCH (http://www.drive5.com/usearch/7.0) was used to analyze the data. Bioinformatics analysis was performed according to the operational taxonomic unit (OTU). Similarity greater than 97% sequence clustering represented an OTU. The ribosomal database project (RDP) classifier was used to systematically classify OTU sequences with reference to the Silva database.

### Protein Extraction and Quality Control

The colon tissue samples were lysed by addition of 600 μl of 8 M urea (lysate: protease inhibitor, 50:1), sonicated for 1 s, stopped for 2 s; this procedure was repeated for a total of 120 s. Samples were centrifuged at 14,000 × g for 20 min at 4°C. Protein was quantified by sodium dodecyl sulphate-polyacrylamide gel electrophoresis. The protein solution rapid prototype high performance liquid chromatograph (RP-HPLC) was separated using an RIGOL L-3000 system (Rigol Technologies, inc.; China) according to the manufacturer’s protocol.

### Peptide Identification by Liquid Chromatography Mass Spectrometry (LC–MS/MS)

The colon tissue samples were lyophilized and ground into powder before being dissolved in 10 µL of 0.1% formic acid solution, centrifuged at 14,000 × g for 20 min at 4°C, then a 1 µg sample was taken for LC–MS/MS measurement. The label-free mass spectrum was analyzed by MaxQuant software and the protein data were screened by Beijing QLBio Company using the Uniprot database.

### Cell Culture

HCoEpiC was cultured in RPMI 1640 (C11875500BT; Gibco) and supplemented with 10% fetal bovine serum (S711-001S; Lonsera) at 37°C in a humidified atmosphere with 5% CO_2_. The following viability experiment was performed in the same manner in all cell lines, with HCoEpiC cells described here as an example. HCoEpiC cells were divided into six groups randomly, marked as control, LPS (model), LD_4_-PDTL, LD_4_-PDTM, LD_4_-PDTH and Dexamethasone (DXMS) groups. All groups were cultured with 2 ml serum-free 1640 medium. Except for the control group, 10 μg/ml LPS was added to each group, and the cells were cultured for another 24 h. The culture medium was replaced with fresh 1640 medium for the control and model groups, while 2 ml serum-free 1640 medium containing 1.9, 3.8, or 7.5 μM LD_4_ was added to LD_4_-PDTL, LD_4_-PDTM and LD_4_-PDTH groups, respectively; 2 ml serum-free 1640 medium containing 7.5 μM DXMS was added to the DXMS positive control group. Cells were cultured with LD_4_ for 30 min. The time was sufficient for bacteria to take up LD_4_, as demonstrated in the bacterial strain deactivation previously reported (Xu et al., 2016; Zhao et al., 2021), so we chose this time to investigate the cellular damage occurring under the same conditions. Cells were irradiated with an energy density of 6 J/cm^2^ or kept in the dark for 30 min, following which the cells were cultured for a further 24 h before MTT assay.

### Quantitative Real-Time PCR Assay

Total RNA in HCoEpiC cells was extracted using the TRIzol Reagent (15596018; Invitrogen) according to the manufacturer’s protocol. qRT-PCR was performed using a UltraSYBR mixture kit (CW0957H; Kangwei Biotech) according to the manufacturer’s instructions. The *IL-1β*, *IL-6*, *TNF-α*, *AOC*_*1*_, *AKT*, and *NF-κB* gene sequences were synthesized by Sangon Biotech (Shanghai, China). Primers are shown in Supplementary Table S1. qRT-PCR was performed using a LightCycler^®^ real-time PCR assay (Roche, China).

### Immunofluorescence Staining

HCoEpiC cells were grown on glass bottomed cell culture dishes (801001; NEST) and treated with LPS for 24 h. HCoEpiC cells were then cultured with LD_4_ for 30 min and irradiated with 650 nm laser light at an energy density of 6 J/cm^2^. Follow-up experiments were conducted at 24 h. Anti-AOC_1_(A6249; ABclonal) and fluorescein-conjugated goat anti-rabbit IgG (ZF-0311; ZSGB-BIO) at 1:100 dilution was added as previously described (Liu et al., 2019).

### Western Blotting Analysis

RIPA (CW2333; CWBio) protein lysate was added to the culture plates containing treated cells as required to extract proteins. According to the molecular weight of the target protein, the corresponding prefabricated glue (C35502009; GenScript) was used, and the loading volume of the protein sample to be tested was 30–60 μg. Electrophoresis was performed at 150 V constant pressure for 50 min. A constant pressure of 100 V was set using the wet rotation method, and the film was transferred by ice bath for 2 h. The membrane was immersed in western blot blocking solution (232100; BD) and shaken gently at room temperature for 2 h. The following rabbit primary antibodies were diluted with TBST and prepared according to the manufacturer’s instructions; *AOC*_*1*_ (16338-1-AP; Proteintech), *AOC*_*1*_ (A6249; ABclonal), *p-NF-κB* (3033; CST), *NF-κB* (8242; CST), p-*IκB* (2859S; CST), *IκB* (4812; CST), *IKK* (2697; CST), *AKT* (4691; CST), *p-AKT* (4060; CST), *p-IKK* (ab178870; Abcam), *IL-6* (WL02841; Wanleibio) and *TNF-α* (WL01581; Wanleibio).

### Cell Transfection

HCoEpiC cells were cultured in six-well plates for 24 h and transfected with short hairpin (sh) RNA (Supplementary Table S2) and overexpressing plasmid vector. Sh-*AOC*_*1*_ inserted into the pGPU6/GFP/Neo vector and the total nucleotide sequence of *AOC*_*1*_ inserted into pEX-1 to obtain the plasmid. All plasmid were synthetized by Gene Pharma (Suzhou, China). Cells were cultured in Opti-mem medium (11058021; Gibco). All transfections were performed using lipofectamine 2000 (11668019; Invitrogen) according to the manufacturer’s protocol.

### Statistical Analysis

Data analysis were conducted using SPSS 18.0 and GraphPad Prism 6. SPSS 18.0 was data statistical analysis software, used to analyze the differences between data. GraphPad Prism 6 was used to plot the corresponding statistics. All data were expressed as mean ± standard deviation (SD). Significant differences were determined using one-way analysis of variance (ANOVA). Statistical differences in 16S rRNA high-throughput sequencing were assessed using Tukey’s HSD (Li H. et al., 2020)^,^ Chen et al., 2020). *p < 0.05* was considered statistically significant.

## Results

### LD_4_-Photodynamic Therapy Reduces Inflammation in LPS-Induced HCoEpiC Cells

**FIGURE 1:**
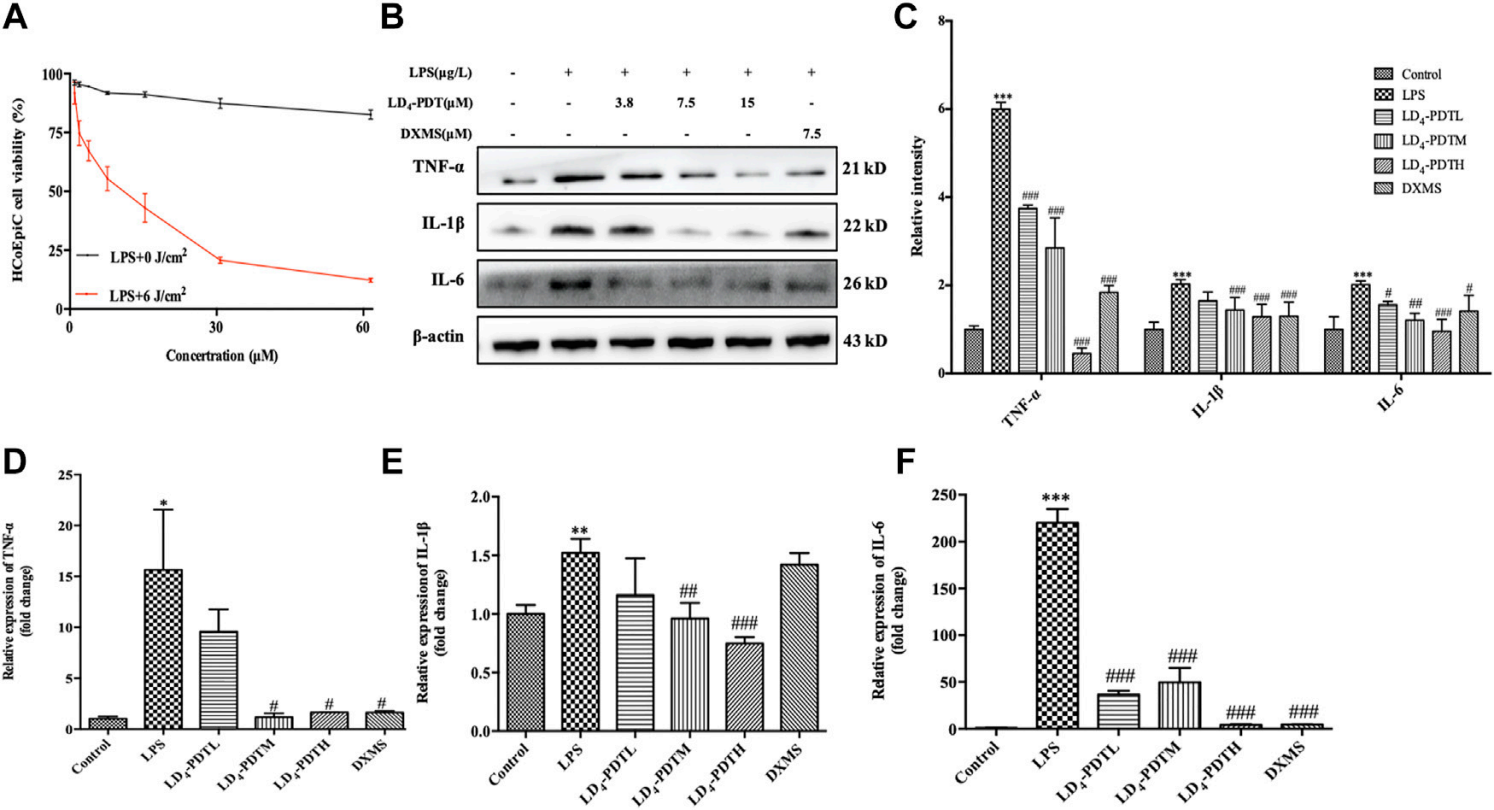
LD_4_-PDT reduced inflammation in LPS-induced HCoEpiC cells. **(A)** HCoEpiC cells were incubated with LPS for 24 h, LD_4_ (0–60 μM) for 30 min, then irradiated (6 J/cm^2^) or untreated. Cell viability was detected by MTT assay. Red line indicated the light reaction; black line indicated the dark reaction. **(B,C)** Protein expression of *TNF-α*, *IL-6* and *IL-1* was determined by western blot. **(D–F)** Expression levels of *TNF-α*, *IL-6* and *IL-1* in HCoEpiC cells were examined by qRT-PCR. ^**^ *p < 0.01*, ^***^ *p < 0.001* vs control group; ^#^ *p < 0.05*, ^##^ *p < 0.01*, ^###^ *p < 0.001* vs LPS model group. Data are representative of three independent experiments, expressed as mean ± SD.

Intestinal epithelial cells were the first protective barrier for the intestinal, its weakness in function would cause the illness in intestinal. So here HCoEpiC cells and LPS-stimulated HCoEpiC cells were chosen as normal and infectious cells *in vitro* model to evaluate the LD_4_-PDT in IBD. DXMS a medicine with efficacy like anti-inflammatory, immunosuppressive and other pharmacological effects, is widely used in the treatment of autoimmune diseases, allergies, inflammation and other diseases in clinics. Therefore, DXMS is often selected as a positive control drug in UC *in vitro* and *in vivo*. Exposure to less than 30 μM LD_4_ in either the dark or the light had no effect on the growth of HCoEpiC cells, indicating that LD_4_-PDT had no obvious toxicity to intestinal tissue cells (Supplementary Figure S1B). While LPS-stimulated HCoEpiC cells were sensitive to LD_4_-PDT, and their proliferation was inhibited in a LD_4_ dose-dependent manner (Figure 1A). Proinflammatory cytokine expression is often changed with the progress of UC. We therefore determined the levels of *IL-6, TNF-α* and *IL-1* in HCoEpiC cells by western blotting and qRT-PCR. The protein and gene expressions of *IL-1*, *IL-6* and *TNF-α* were significantly decreased in the LD_4_-PDT-treated group compared with control group (Figures 1B–F).

### LD_4_-Photodynamic Therapy Treatment of TNBS-Induced UC Model Rats

**FIGURE 2:**
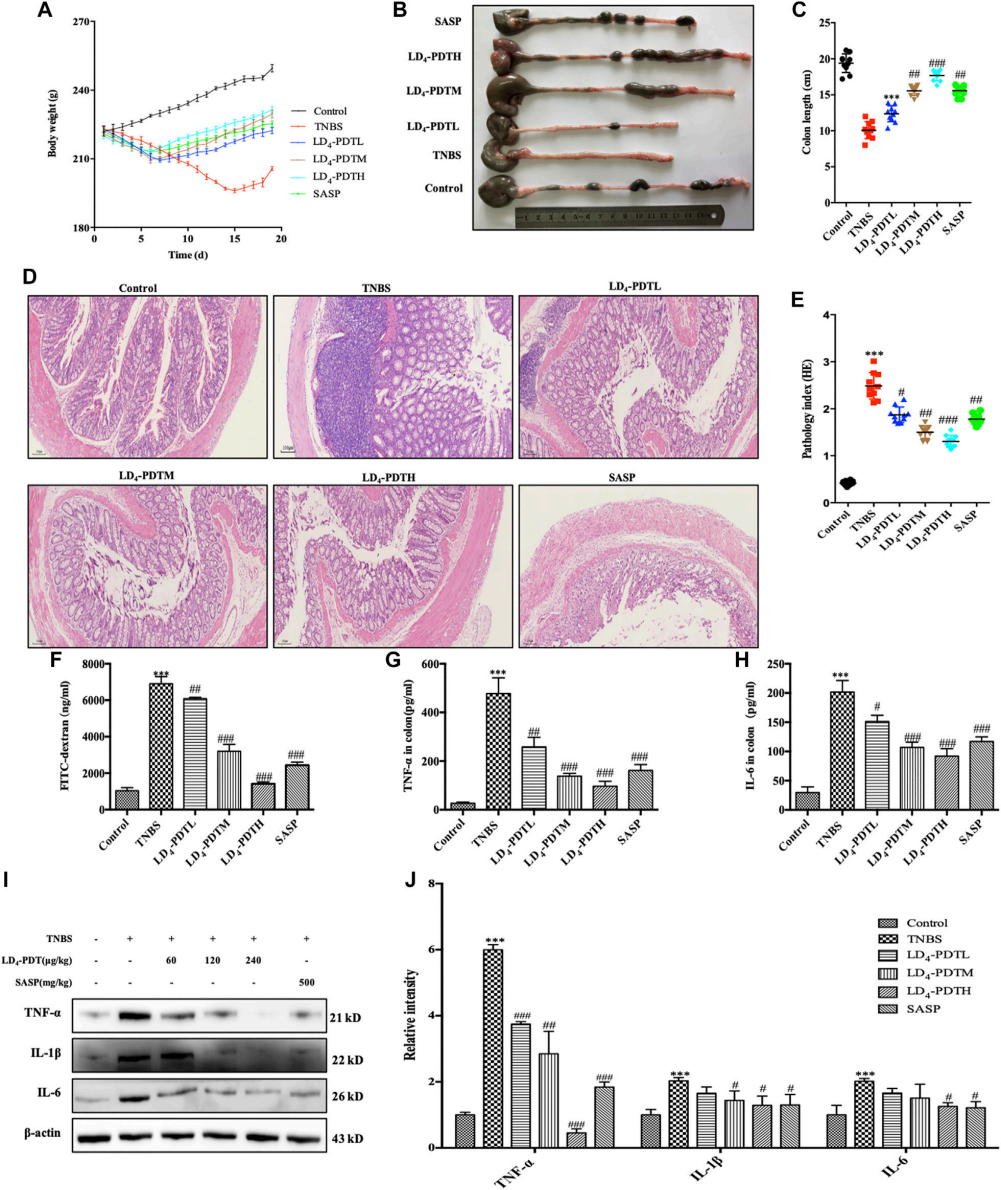
LD_4_-PDT alleviated TNBS-induced UC in model rats. **(A)** Body weight of UC model rats changed with time. **(B,C)** Length of colon. **(D,E)** Hematoxylin and eosin staining of colon tissue and inflammation score. **(F)** Serum fluorescent intensity of FITC-dextran. **(G,H)** *TNF-α* and *IL-6* levels determined by ELISA. **(I,J)** Protein expression of *TNF-α*, *IL-6* and *IL-1* were determined by western blot analysis. Each column represents the mean ± SD of 10 rats per group. ^**^ *p < 0.01*, ^***^ *p < 0.001* vs control group; ^#^ *p < 0.05*, ^##^ *p < 0.01*, ^###^ *p < 0.001* vs TNBS-treated group.

Our previous research has shown that LD_4_ could inactivate microbial pathogens in traumatic infection ((Meng et al., 2015; Xu et al., 2016; Zhao et al., 2021). We have proved that the three different doses of LD_4_ used in this paper have no significant effect on normal rats and PDT alone did not significantly relieve UC (Supplementary Figure S1F,G). The occurrence and development of IBD is also closely related to the change of gut microbes. On the basis of these results, we suggested LD_4_-PDT could be used to treat UC. UC model was established using TNBS, and the treatment was started from day 7 post-induction, LD_4_ at doses of 60, 120, and 240 μg/kg was administrated *via* enema every second day, 30 min later after which the colon was irradiated with an intensive 650 nm PDT system at an energy density of 25 J/cm^2^, SASP, a standard UC drug as a positive control, was administered to the control group. The treatment was preformed four times. A steady increase in body weight in control group and body weight decrease in TNBS model group was observed, while body weight increasing in LD_4_-PDT or SASP treated rats (Figure 2A). After LD_4_-PDT treatment the animals were sacrificed and the colon was harvested. Its inner wall in the normal control group was complete, with regular folds and clear vascular texture, no obvious erosion, ulcers, or granuloma. In the TNBS model group, the colon intestine was shortened, the mucosa was marked with hyperemia and edema, scattered erosion or ulcers with hemorrhage, and large ulcerated areas. While the length of the colon and the thickness of the intestinal wall were improved to varying degrees in LD_4_-PDT-treated or SASP control groups (Figures 2B,C). Colon histopathology showed that in TNBS group, epithelial cells shedding off, inflammatory cell infiltration in the sub-membrane, crypt abscess and ulcer formation were observed locally, mucosal glands were disorganized, destroyed or absent, goblet cells were reduced or absent, and histological injury score was significantly increased. Compared with the model group, LD_4_-PDT-treated rats showed significantly alleviated pathological symptoms in the colon, and the colonic mucosal glands were arranged neatly, with few edemas and almost no ulcerative exudate (Figures 2D,E). The intestinal epithelial barrier is an important part of the intestinal innate immunity, and so measuring the permeability of the intestinal wall could determine disease activity. Following administration of FITC-dextran by intragastric gavage, the fluorescence intensity in serum indirectly reflects the intestinal permeability, the higher fluorescent intensity, the more severe the intestinal damage. Results showed that the levels of FITC-dextran in the serum of normal control rats were very low but significantly increased in TNBS groups, indicating that intestinal permeability was increased, and the intestinal wall was damaged following TNBS exposure. The content of FITC in serum of LD_4_-PDT- and SASP-treated groups was significantly decreased, indicating that these drugs had a protective effect on intestinal integrity (Figure 2F). In accordance with the results above, the expression of *IL-1*, *IL-6* and *TNF-α* either in serum or in colon tissue were lower in LD_4_-PDT-treated rats compared with the TNBS group (Figures 2G–J).

### LD_4_-Photodynamic Therapy Regulates Oxidative Stress in TNBS-Induced UC Model Rats

**FIGURE 3:**
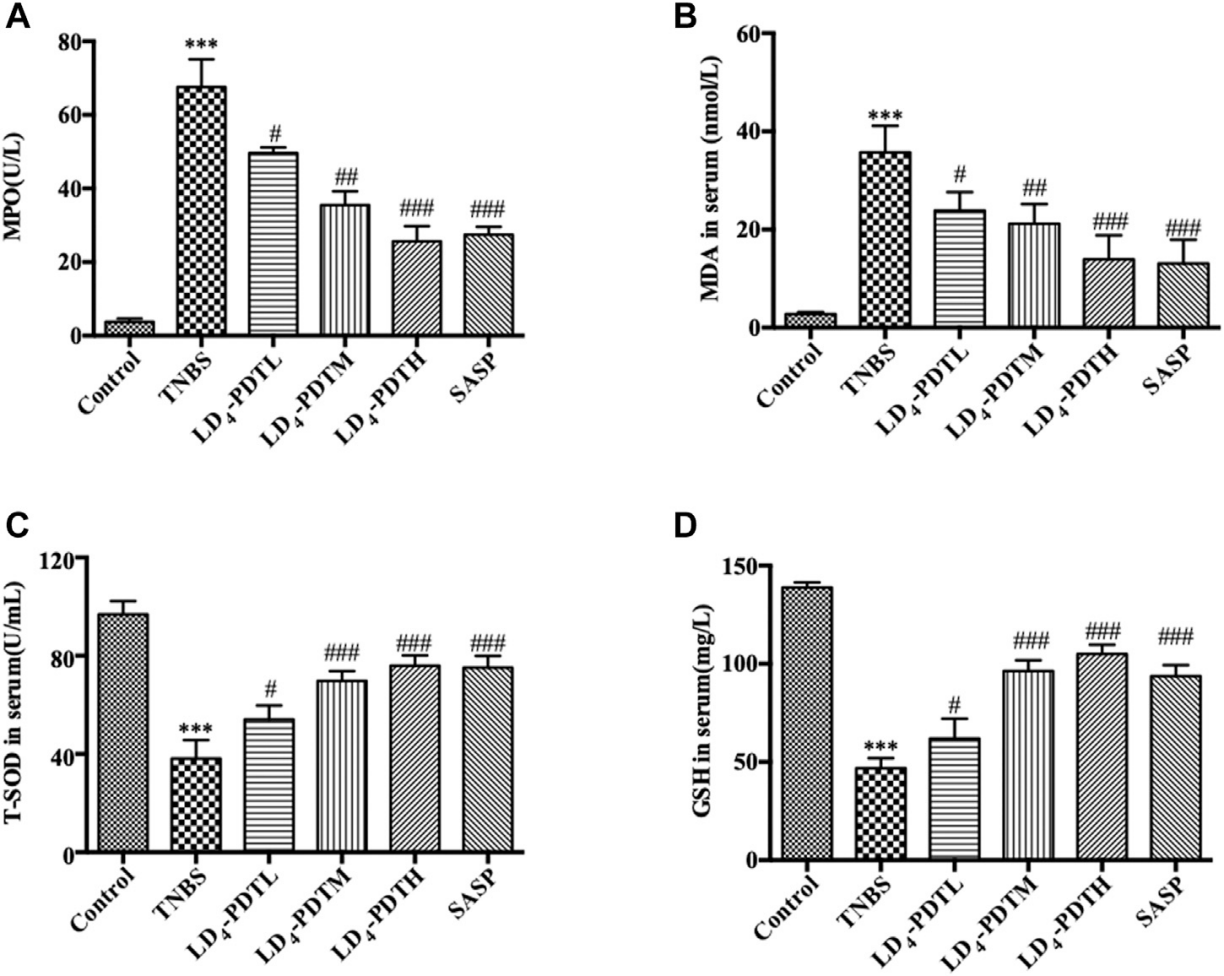
LD_4_-PDT regulated oxidative stress in TNBS-induced UC model rats. Concentrations of **(A)** *MPO*, **(B)** *MDA*, **(C)** *SOD* and **(D)** *GSH* in rat’s serum were examined by ELISA. **p < 0.01, ***p < 0.001 vs control group; ^#^ *p < 0.05*, ^##^ *p < 0.01*, ^###^ *p < 0.001* vs TNBS model group. *n* = 10 rats per group.

Because there is a close relationship between oxidative stress and UC (Morris et al., 1989), the level of *MPO* and *MDA* in serum as indicators of oxidative stress were investigated. *MPO* and *MDA* were significantly increased in the TNBS group but decreased in LD_4_-PDT-treated rats in a dose dependent manner (Figures 3A,B), although levels in these animals remained higher than those of the normal control rats. Furthermore, the levels of two antioxidants *GSH* and *SOD* in serum were significantly decreased after TNBS exposure but increased significantly after LD_4_-PDT treatment (Figures 3C,D). These changes all indicated that the LD4-PDT treatment could improve the living state of UC rats.

### LD_4_-Photodynamic Therapy Reprograms the Protein Profile of Colon Tissues in TNBS-Induced UC Model Rats

**FIGURE 4:**
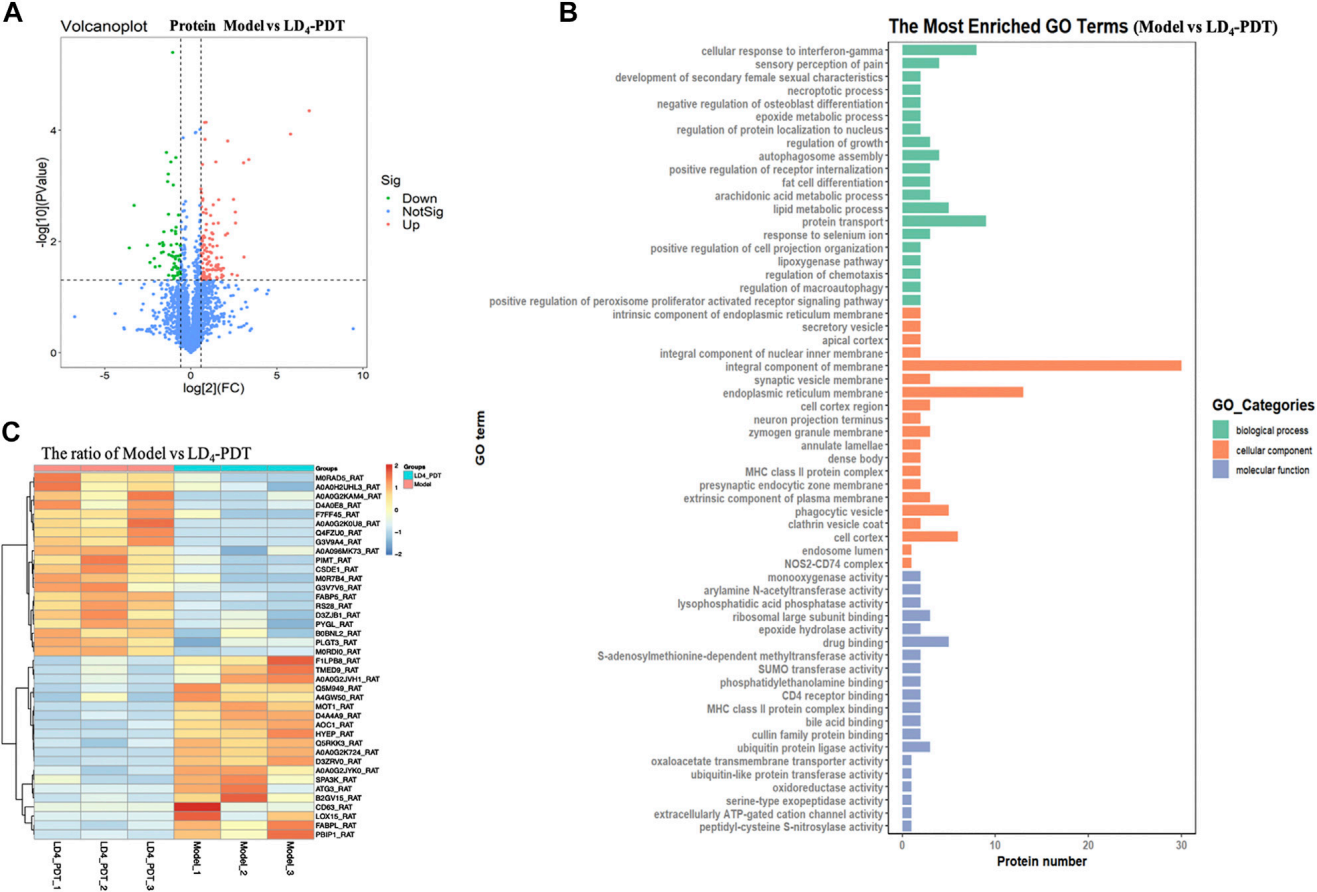
LD_4_-PDT reprogrammed the protein profile of colon tissues in TNBS-induced UC model rats. **(A)** Volcano plots showing the difference in protein expression among model and LD_4_-PDT groups. **(B)** All identified proteins were classified according to the first 20 gene ontology ratios sorted by enrichment degree [−log10 (*p-value*)] and protein number according to biological process, cell composition and molecular function. Over-representation analysis was used for functional enrichment analysis, and the t-test based on hypergeometric distribution. **(C)** Heat map of 40 differentially expressed proteins among the model and LD_4_-PDT groups. Each column represented a sample, and each row represented a factor; red indicated elevated protein expression, green indicated decreased protein expression.

In order to investigate the mechanism of LD_4_-PDT in the treatment of UC, the related protein profile was analyzed based upon proteomics. Label-free proteomics was used to detect the protein profiles in colon tissues of rats in the control, TNBS model and LD4-PDT treatment groups. A *t*-test was used directly for differential analysis, and the differentially expressed proteins meeting the criteria of *p ≤ 0.05* and fold change ≥1.5 times were screened. Scatter plots showed changes in protein expression between the TNBS model and LD_4_-PDT groups (Figure 4A). Compared with LD_4_-PDT-treated rats, there were 176 differentially expressed sites in the TNBS model group, among which 116 were highly expressed (Figure 4A, red) and 60 showed low expression (Figure 4A, green). Differentially expressed proteins are shown in Supplementary Figure 2A for comparison with other groups. Gene ontology (GO) is a standard vocabulary to describe the function, location and activity of genes, which covered three aspects of biology, namely biological process, cellular component, and molecular function. GO analysis of biological processes showed that differentially expressed proteins between the control and TNBS model groups were significantly related to collagen fibril organization, collagen biosynthesis, peptide acetyl threonine phosphorylation and negatively regulated biosynthesis by cell adhesion (Supplementary Figure S2B). However, treatment with LD_4_-PDT changed the colonic protein expression profiles (Figure 4B). In terms of cellular component, the differentially expressed proteins were related to intracellular organelles, collagen and immunoglobulin complexes (Supplementary Figure S2B), indicating that treatment with LD_4_-PDT resulted in changes in the cell cortex, intracellular organelles, and major histocompatibility complex class II proteins (Figure 4B). In terms of molecular function, the differentially expressed proteins were mainly derived from structural components of the extracellular matrix and the activities of ligase and transferase (Supplementary Figure S2B), while treatment with LD_4_-PDT changed the activities of oxygenase and hydrolase (Figure 4B). We screened 40 significantly different proteins using heat maps to illustrate the differing protein expression profiles in the colon samples. The results showed that expression of *AOC*_*1*_ in model rats was obviously increased (Supplementary Figure S2C), while this upregulation was eliminated following treatment with LD_4_-PDT (Figure 4C). The role of *AOC*_*1*_ in immune cell transport made it a target for autoimmune and inflammatory diseases, so we selected *AOC*_*1*_ for further study (Peet et al., 2011).

### LD_4_-Photodynamic Therapy Suppresses the Expression of *AOC*_*1*_/*AKT*/*IKK*/*NF-κB in vitro and in vivo*

**FIGURE 5:**
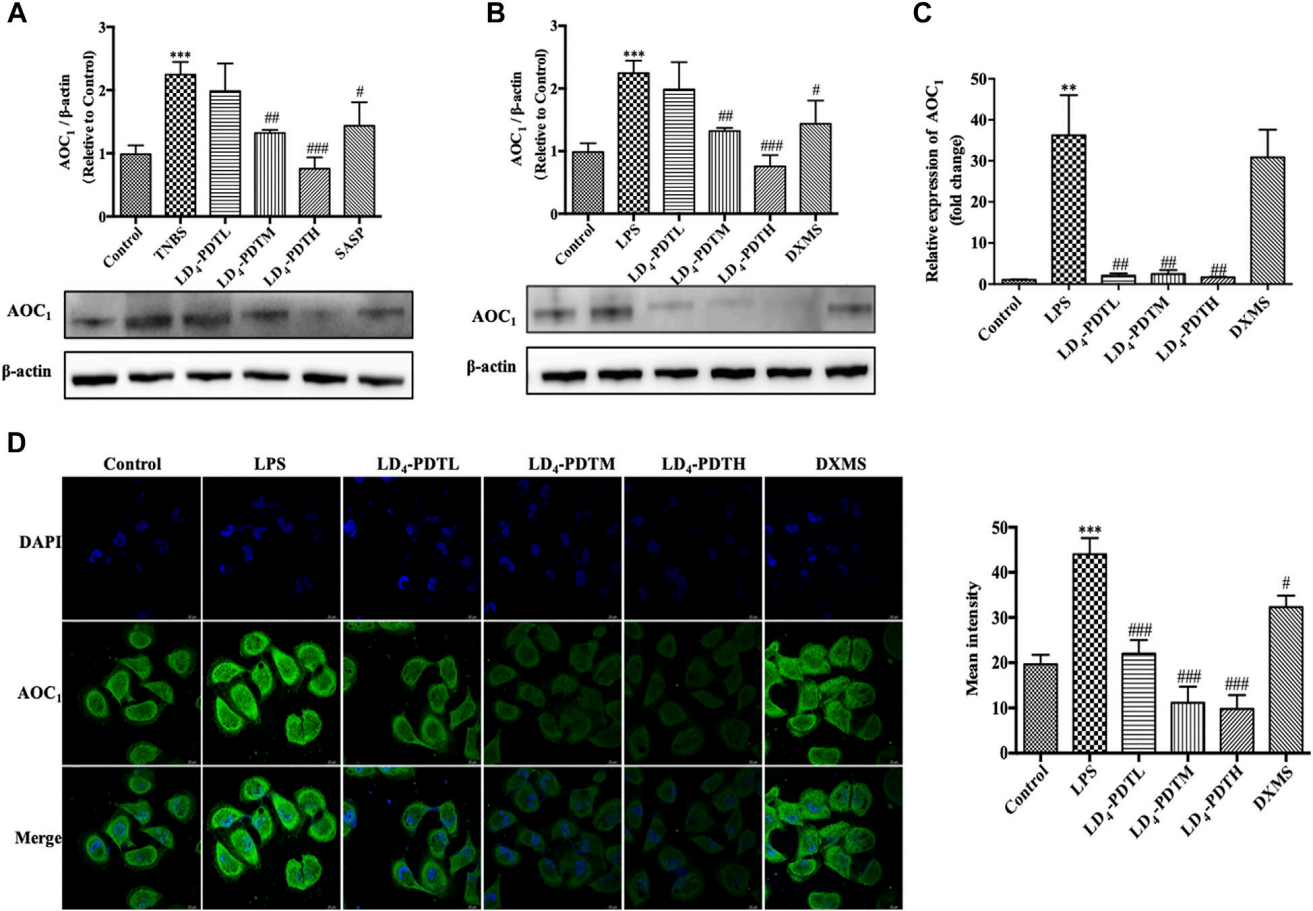
LD_4_-PDT suppressed the expression of *AOC*_*1*_. **(A,B)** Protein expression of *AOC*_*1*_ in colon tissue and in HCoEpiC cells were determined by western blot analysis. **(C)** Gene expression of *AOC*_*1*_ in HCoEpiC cells was examined by qRT-PCR. **(D)** Protein expression of *AOC*_*1*_ was detected by immunofluorescence. Bars, 20 μm **p < 0.01, ***p < 0.001 vs control group; ^#^ *p < 0.05*, ^##^ *p < 0.01*, ^###^ *p < 0.001* vs TNBS model group. Data were representative of three independent experiments, expressed as mean ± SD.

**FIGURE 6:**
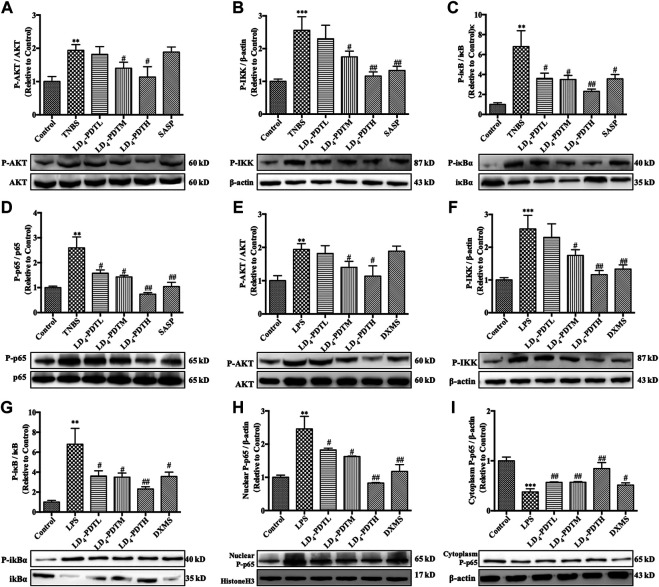
LD_4_-PDT inhibited the expression of *AOC*_*1*_/*AKT*/*IKK*/*NF-κB*. **(A–D)** Protein expression of **(A)** *p-AKT*, **(B)** *p-IKK,* **(C)** *p-IκB* and **(D)** *p-p65* in colon tissue were determined by western blot. **(E–I)** Protein expression of **(E)** *p-AKT*, **(F)** *p-IKK*, **(G)** *p-IκB*, **(H)** *p-p65* (in nuclear), and **(I)** *p-p65* (in cytoplasm) in HCoEpiC cells were determined by western blot **p < 0.01, ***p < 0.001 vs control group; ^#^ *p < 0.05*, ^##^ *p < 0.01*, ^###^ *p < 0.001* vs TNBS model or LPS model group. Data are representative of three independent experiments, expressed as mean ± SD.

Western blotting was used to further verify the expression of *AOC*_*1*_ protein. Compared with the control group, the expression of *AOC*_*1*_ was significantly increased in the TNBS model group but was significantly decreased after LD_4_-PDT treatment (Figure 5A). The expression of *AOC*_*1*_ in HCoEpiC cells was detected by western blot (Figure 5B), qRT-PCR (Figure 5C) and immunofluorescence (Figure 5D). Consistent with the results of the animal experiments, *AOC*_*1*_ protein and gene expression were up-regulated after LPS treatment in HCoEpiC cells but were then reduced upon LD_4_-PDT treatment. Because *AOC*_*1*_ can activate *AKT* and downstream pathways (Xu et al., 2020), the key factors interacting with *AOC*_*1*_ were screened. Western blotting showed that compared with the control group, the expression of *p-AKT*, *p-NF-κB*, *p-IKK,* and *p-IκB* was increased significantly in the TNBS group, while LD_4_-PDT treatment significantly decreased the expressions of *p-AKT*, *p-NF-κB*, *p-IKK*, and *p-IκB* compared with the TNBS model group (Figures 6A–D). Similarly, the expressions of *p-AKT*, *p-NF-κB*, *p-IKK,* and *p-IκB* were significantly decreased after LD_4_-PDT treatment compared with LPS stimulation alone in HCoEpiC cells (Figures 6E–I). Thus, our findings suggest that treatment with LD_4_-PDT may decrease or block the expression of *AOC*_*1*_ */AKT*/*IKK*/*NF-κB* in UC.

### LD_4_-Photodynamic Therapy Protects Intestine *via* Inhibition of *AOC*_*1*_

**FIGURE 7:**
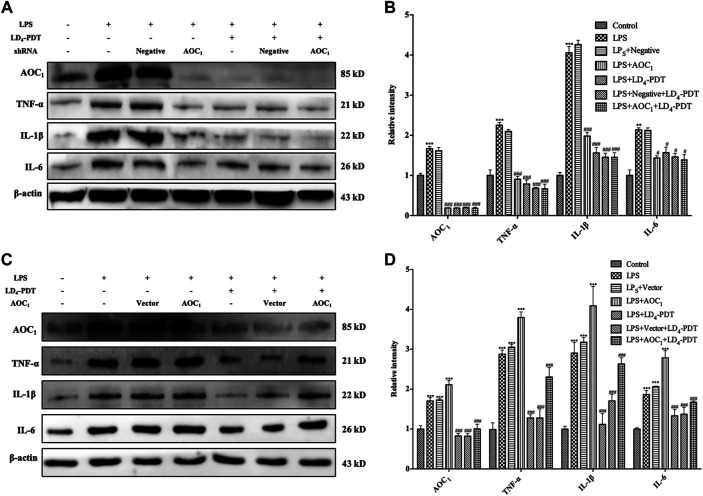
LD_4_-PDT protected intestine *via* inhibition of AOC_1_. **(A,B)** LD_4_-PDT effect on *TNF-α*, *IL-6* and *IL-1* expression in *AOC*_*1*_ knockdown HCoEpiC cells. **(C,D)** LD_4_-PDT effect on *TNF-α*, *IL-6* and *IL-1* expression in *AOC*_*1*_ over-expression cells. **p < 0.01, ***p < 0.001 vs control group; ^#^ *p < 0.05*, ^##^ *p < 0.01*, ^###^ *p < 0.001* vs LPS groups. Data are representative of three independent experiments, expressed as mean ± SD.

The above results indicated that *AOC*_*1*_ played an important role in LD_4_-PDT treatment of UC, so the impact of *AOC*_*1*_ protein in LD_4_-PDT treatment was further investigated. We knocked down the *AOC*_*1*_ gene in HCoEpiC cells using a specific shRNA then induced the inflammatory model with LPS before treating the cells with LD_4_-PDT. Western blot verified that more than 70% AOC_1_ was silenced (Supplementary Figure S3A), and knockdown *AOC*_*1*_ led to reduce the levels of *IL-1*, *IL-6* and *TNF-α* in the cells to a varying degree compared with mock-transfected cells. Following LD_4_-PDT treatment, the expression levels of these cytokines in all groups was reduced remarkably, and there was no obvious difference among knockdown *AOC*_*1*_ group and other group (Figures 7A,B). On the contrary, overexpressed *AOC*_*1*_ cells was also built, the same treatment was conducted as that of the down-regulated cells (Supplementary Figure S3B). Western blot assay showed that compared with mock-transfected cells, protein levels of IL-1, IL-6 and *TNF-α* in the *AOC*_*1*_ overexpression cells were increased, while these cytokines all reduced following LD_4_-PDT treatment in all cells. Among all LD_4_-PDT treatment cells, *AOC*_*1*_ overexpression HCoEpiC cells exhibited the higher protein levels of *IL-1, IL-6* and *TNF-α* (Figures 7C,D). We set AOC_1_-knockdown group and AOC_1_-overexpression group in HCoEpiC cells without LPS stimulation, as well as control cells group, to examine whether AOC_1_ is critical for cytokine production at the basal conditions. Our results demonstrated that AOC_1_ plays an important role in basal condition (Supplementary Figure S3C,D).Summary above, we found that the expression of *AOC*_*1*_ was up-regulated in the model group, while LD_4_-PDT treatment reduced the expression of *AOC*_*1*_ at the gene and protein levels, indicating that LD_4_-PDT exerted its effects in UC *via AOC*_*1*_, thus mediating expression of downstream cytokines. These results suggested that LD_4_-PDT treatment may work *via* inhibition of *AOC*_*1*_ to mediate *AKT/IKK/NF-κB* pathways, and thus knockdown of *AOC*_*1*_ protein either by LD_4_-PDT treatment or other means could alleviate UC symptoms.

### LD_4_-Photodynamic Therapy Remodels the Gut Bacterial Composition Pattern in TNBS-Induced Ulcerative Colitis Model Rats

**FIGURE 8:**
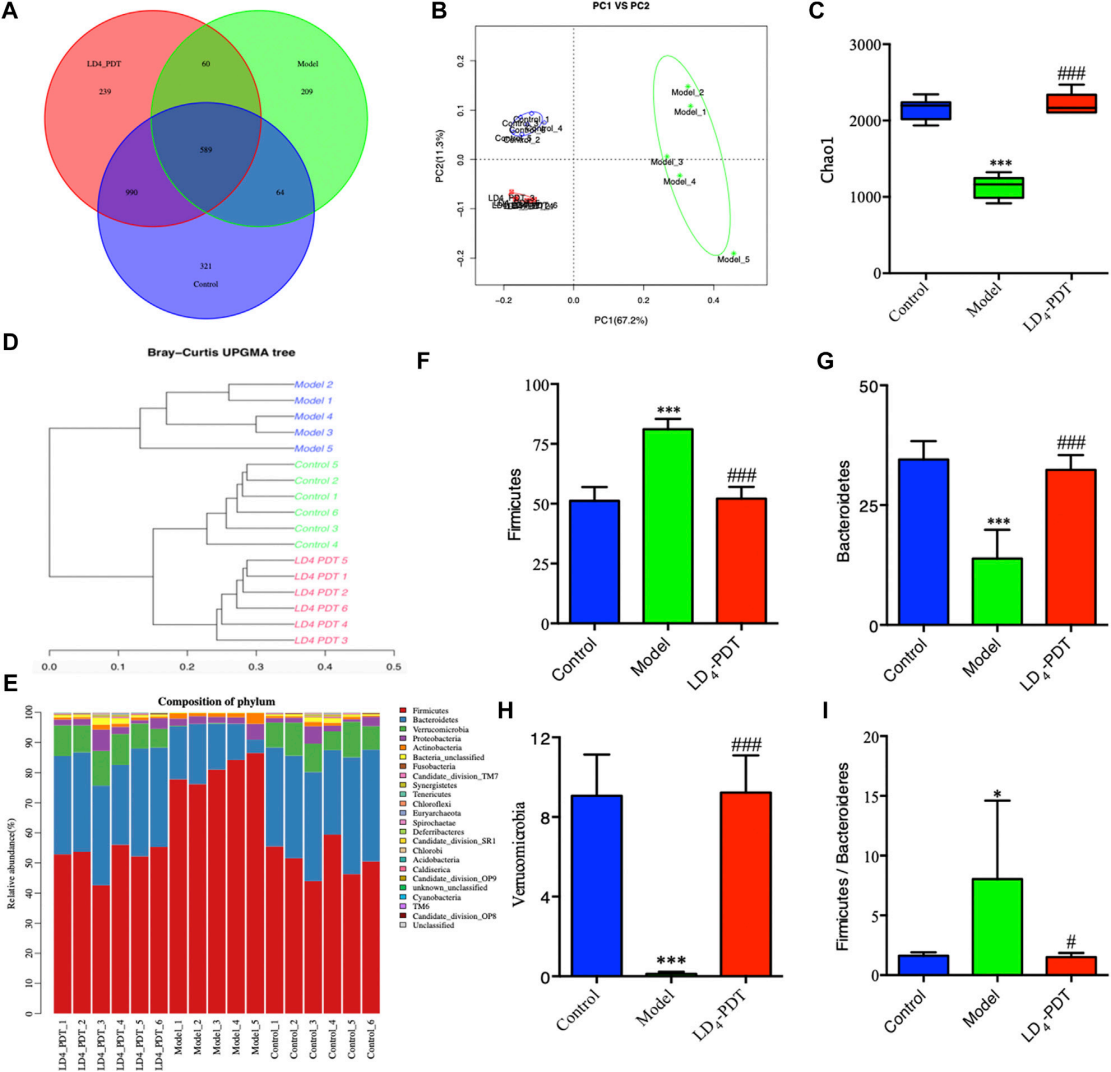
LD_4_-PDT treatment altered the gut microbiota in TNBS-induced UC model rats. **(A)** Venn diagram indicated the differential numbers of OTUs in each group. **(B)** PCoA score. **(C)** Chao1 index represented the *α*-diversity of the gut microbiota. **(D)** Hierarchical clustering of fecal microbiota in the control, model and LD_4_-PDT groups. **(E)** Phylum-level distribution of fecal microbiota **(F–H)** Relative abundance of the **(F)** Firmicutes, **(G)** Bacteroidetes, **(H)** Verrucomicrobia **(I)** The Firmicutes/Bacteroidetes (F/B) value **p < 0.01, ***p < 0.001 vs control group; ^#^ *p < 0.05*, ^##^ *p < 0.01*, ^###^ *p < 0.001* vs model group; *n* = 6 rats per group.

Effect of LD_4_-PDT on the gut microbes among the control, TNBS and LD_4_-PDT-treated groups were studied using the 16S rRNA method. The Venn diagram (Figure 8A) shows that a total of 2472 OTUs were obtained from all samples. A total of 589 OTUs were appeared in all three groups, while 990 OTUs were shared between the control and LD_4_-PDT groups, 64 OTUs were shared between the TNBS model and control groups, the TNBS model and LD_4_-PDT groups shared 60 (Figure 8A). The Shannon curve showed that sequencing depth covered rare new phylotypes, with both diversity and Shannon index in the TNBS model group being lower than those in the control and LD_4_-PDT groups (Supplementary Figure S4A). Weighted UniFrac-based principal coordinates analysis (PCoA) indicated the unique intestinal microbiota composition clustering of individual groups (Figure 8B). The *α*-diversity of intestinal bacteria decreased significantly in TNBS model rats, but it was recovered to an almost normal state after LD_4_-PDT treatment (Figure 8C, Supplementary Figure S4B). Unweighted pair group method with arithmetic mean analysis showed that there was a significant difference in intestinal flora between the TNBS model group and LD_4_-PDT-treated rats, and LD_4_-PDT treatment made the overall composition of intestinal flora in UC model rats return to a state similar with the control group (Figure 8D). Taxonomic bins at the phylum level indicated that gut bacterial composition patterns in model animals were obviously different from the control groups (Figure 8E): the proportions of Firmicutes in fecal stool of rats in the TNBS model group were higher than those in the normal control group (Figure 8F), while the abundance of Bacteroidetes (Figure 8G) and Verucomicrobia (Figure 8H) were lower than in control rats, the Firmicutes/Bacteroidetes (F/B) value in the TNBS model group was higher than that of the control group (Figure 8I); however, LD_4_-PDT treatment reduced Firmicutes, increased Bacteroidetes and Verucomicrobia in fecal samples, the F/B value was reduced after LD_4_-PDT treatment, and the gut bacterial composition pattern was comparable with that of the control group following LD_4_-PDT treatment (Figures 8F–I).

## Discussion

In this study, we found that LD_4_-PDT can effectively alleviate the inflammation both *in vitro and in vivo.* TNBS-induced UC model rats showed ruffled fur, loss of appetite, lethargy, blood in the stool, and weight loss, while LD_4_-PDT treatment recovered their healthy appearance with no significant weight loss. Pathological observations revealed that the colonic mucosal barrier structure of the untreated group was destroyed with infiltration of inflammatory cells and obvious intestinal wall ulcers and adhesions, these illness characteristics was reduced in each LD_4_-PDT-treated group. Neutrophils are rich in *MPO*, which can catalyze and oxidize chloride ions to produce hypochlorous acid to kill microorganisms in phagocytes, destroy a variety of target substances, and play a role in production and regulation of inflammation (Gu et al., 2017). *MDA* levels can reflect the degree of external damage to the body (Wang et al., 2019). Neutrophils in colitis model release *MPO,* which stimulates oxidative stress in the body, and increases the level of *MPO* and *MDA*. However, LD_4_-PDT treatment significantly reduced both *MPO* and *MDA* levels. *SOD* which can remove the harmful substances produced in the process of metabolism (Gu et al., 2017; Wang et al., 2019), is the only enzyme that decomposes superoxide radicals into H_2_O_2_ in antioxidation. Another free radical scavenger, *GSH*, can also regulate oxidative stress (Gu et al., 2017; Wang et al., 2019). The levels of *GSH* and *SOD* were decreased in the colitis model, but were increased significantly after LD_4_-PDT treatment, indicating that LD_4_-PDT can regulate the level of oxidative stress during UC.

The inhibitory effect of amino salicylic acid drugs on *NF-κB* can improve the symptoms of UC (Murray et al., 2020). The role of *TNF-α* is mainly to regulate the function of immune cells and induce inflammation to produce *IL-1* and *IL-6.* Many studies have shown that the levels of *TNF-α, IL-1* and *IL-6* were elevated in the UC model (Wang et al., 2019; Zhai et al., 2020), while further studies have confirmed that inhibition of *TNF-α, IL-1* and *IL-6* could prevent the development of inflammation (Xu et al., 2017; Lee et al., 2020). In this study, LD_4_-PDT treatment significantly inhibited *TNF-α*, *IL-1* and *IL-6* expression, and consequently reduced the severity of colitis in UC model (Huang et al., 2017; Lee et al., 2020; Li et al., 2020). Intestinal barrier function can be assessed by quantification of permeability, so fluorescent tracers can be used to detect the degree of permeability by measuring the fluorescence intensity in serum. Many studies have shown that in UC models, the intestinal mucosal barrier function is impaired and so intestinal permeability increases, allowing harmful substances to invade the body and promoting development of the disease (Wang et al., 2019; Li H. et al., 2020). After LD_4_-PDT treatment, the intestinal permeability was decreased and intestinal mucosal barrier function was recovered. The above results show that LD_4_-PDT can not only restore the intestinal mucosal barrier function, but also reduce the damaging effects of mucosal injury factors on the intestinal mucosa, thus playing a key role in the treatment of UC.

The intestinal flora is the largest and most complex microecosystem in the human body. It is a dynamic community composed of bacteria, fungi, and viruses that regulate the homeostasis and physiological functions of the mucous membrane. There is a relatively balanced state between the body’s intestinal microbes and the immune system. Abnormal immune responses to bacteria out of balance may disrupt this homeostasis and are related to human IBD (Le Chatelier et al., 2013). Several studies have shown that intestinal flora affects intestinal mucosal immune function, intestinal cell metabolism and renewal and other processes, which can cause obesity, diabetes, malignant tumors, toxemia and IBD (Chen et al., 2020; Ma et al., 2020; Wang et al., 2020). Therefore, exploring the role of the intestinal flora in the occurrence and development of UC, and restoring the balance of disordered flora is of great significance for the treatment of UC. 16S rRNA sequencing technology has shown that 90% of the dominant human intestinal flora consists of Firmicutes, Bacteroidetes, Proteobacteria and Verrucomicrobia (Nishino et al., 2018; Soltys et al., 2020; Wang et al., 2020; Zhu et al., 2020). Both UC patients and UC mice have serious flora imbalances, and the distribution of UC flora at different stages of disease is also significantly different. In our study, we found that Firmicutes and Bacteroidetes were dominant in the model group, followed by Verrucomicrobia. LD_4_-PDT treatment increased the diversity of the flora and restored the flora composition towards normal. The F/B value, the ratio of the abundance of Firmicutes and *Bacteroides*, can effectively reflect the disorder of the intestinal flora and is usually significantly increased in the pathogenesis of UC (Wu et al., 2019; Zhu Y. et al., 2020). In our study, the F/B value in the TNBS model group was higher than that of the control group, and the F/B value was reduced after LD_4_-PDT treatment, suggesting that LD_4_-PDT can improve intestinal diseases to a certain extent. Verrucomicrobia, which gain energy from degradation of excess mucin produced in the lining of the gut and produce anti-inflammatory effects, are significantly more abundant in healthy persons than in patients with UC (Shah et al., 2016; Zakerska-Banaszak et al., 2021). In UC patients, the level of mucin is decreased resulting in reduced abundance of Verrucomicrobia (Shah et al., 2016; Zakerska-Banaszak et al., 2021). Consistent with previous reports, we found that at the phylum level, the relative content of Verrucomicrobia was reduced in the UC model compared with control rats, while LD_4_-PDT treatment increased the abundance of this microbiota and protected the intestinal mucosa to produce an anti-inflammatory effect.

To further explore the biological mechanism of LD_4_-PDT in UC, colonic protein assays were conducted using proteomics in model, control and LD_4_-PDT groups. More than 176 proteins were detected and their differential expression were given between model and LD_4_-PDT group. Although the first two protein changed large, however they were related to basic immunology and energy supply. AOC_1_, whose abundance change was in third position and was correlated tightly with the inflammation, were identified as a potential target of LD_4_-PDT. AOC_1_ is known to be involved in allergic and immune responses, cell proliferation, tissue differentiation, and apoptosis (Strolin Benedetti et al., 2007; Shepard and Dooley, 2015; Vakal et al., 2020). Its role in the transport of immune cells has made it a target for autoimmune and inflammatory diseases (Peet et al., 2011). In addition, *AOC*_*1*_ has the ability to regulate pathophysiological processes, such as cancer and EMT (Xu et al., 2020). Studies have reported that plasma *AOC*_*1*_ activity can be used to diagnose intestinal integrity and play an important role in UC (DiSilvestro et al., 1997). In this study, we found that the expression of *AOC*_*1*_ in the colon was up-regulated in the TNBS model group, while LD_4_-PDT treatment reduced the expression of *AOC*_*1*_ at the gene and protein levels, indicating that LD_4_-PDT exerted its effects in UC *via AOC*_*1*_ *,* thus mediating expression of downstream cytokines. To test this hypothesis, *AOC*_*1*_ was specifically knocked down or over-expressed *in vitro*. Consistent with our hypothesis, silencing of AOC_1_ reduced the levels of *IL-1, IL-6* and *TNF-α*. These results suggested that *AOC*_*1*_ protein may be a key protein in the treatment of UC, and that reducing its expression, either by LD_4_-PDT treatment or by other means, could ameliorate UC symptoms. Studies have shown that *AOC*_*1*_ can promote the expression of *AKT* and further regulate pro-inflammatory factors like *NF-κB* (Xu et al., 2020). The activated *NF-κB* signaling pathway can affect various biological processes, including innate and adaptive immunity, inflammation, stress responses, and B-cell development (Liu et al., 2020; Shamekhi et al., 2020). The AOC_1_-AKT/IKK/ NF-κB pathway plays an important role in immune response and inflammatory response and is involved in the occurrence and development of various inflammatory diseases. Activation of NF-κB can promote the expression of various inflammatory factors such as IL-6, LI-1*β* and TNF-*α*, enhance the body’s non-specific and specific immune responses, cause tissue damage and organ dysfunction, and further aggravate the symptoms of UC. Our research shows that when the UC model was established, the *AKT/IKK/NF-κB* pathway was activated to promote inflammation but following LD_4_-PDT treatment, the *AKT/IKK/NF-κB* pathway was inhibited, thereby exerting a therapeutic effect on UC. In summary, our study indicates that LD_4_-PDT treatment UC was effective, the working mechanism may be LD_4_-PDT function *via* AOC_1_ to mediate *AKT/IKK/NF-κB* pathways and downstream inflammatory cytokine expression. However, the mechanism of action behind LD_4_-PDT function and whether AOC_1_ is the sole target of LD_4_-PDT in the treatment of UC remain to be fully elucidated.

In conclusion, we have shown that LD_4_-PDT treatment could promote healing of the colonic mucosa, regulation of intestinal flora, and improvement in the clinical symptoms of UC. LD_4_-PDT can reduce the mucosal inflammatory response mediated by AOC_1_, which we identified as a potential target for UC intervention. Novel photosensitizing agent, LD_4_, was an efficient PDT treatment candidate for UC.

## Data Availability

The original contributions presented in the study are publicly available. This data can be found here: http://proteomecentral.proteomexchange.org/cgi/GetDataset, submission number - 1-20210807-77236.
