# Supplementary material for: Photodynamic Therapy of Novel Photosensitizer Ameliorates TNBS-Induced Ulcerative Colitis via Inhibition of AOC1
Source: Front Pharmacol. 2021 Oct 21;12:746725. doi: 10.3389/fphar.2021.746725 (PMC8566348; doi:10.3389/fphar.2021.746725)
Supplement: Supplementary file 1 [file DataSheet1.docx]

Photodynamic therapy of novel photosensitizer ameliorates TNBS-induced ulcerative colitis via inhibition of AOC_1_ Supplementary Figures

**Supplementary Figures**

**
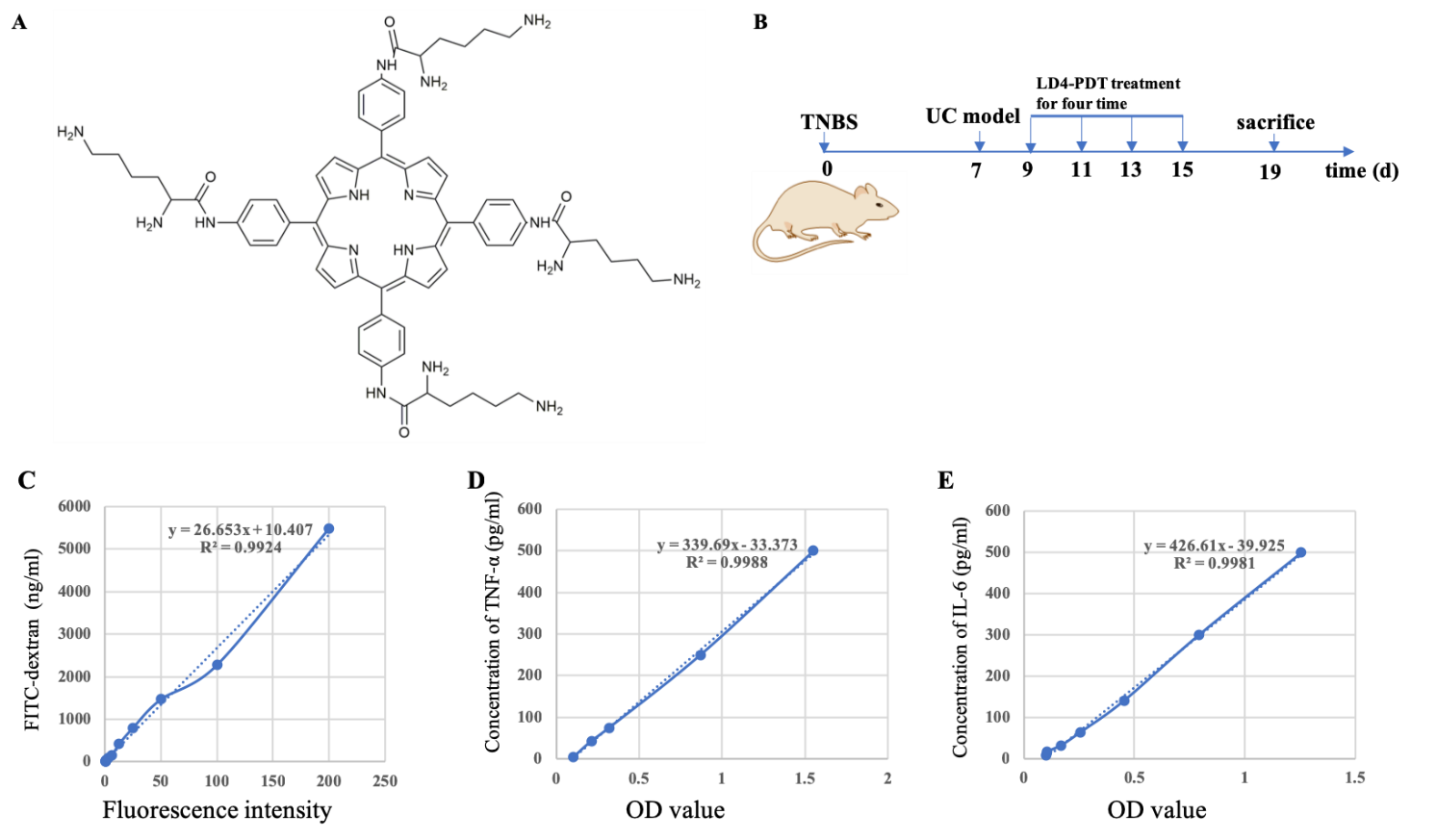
**

**
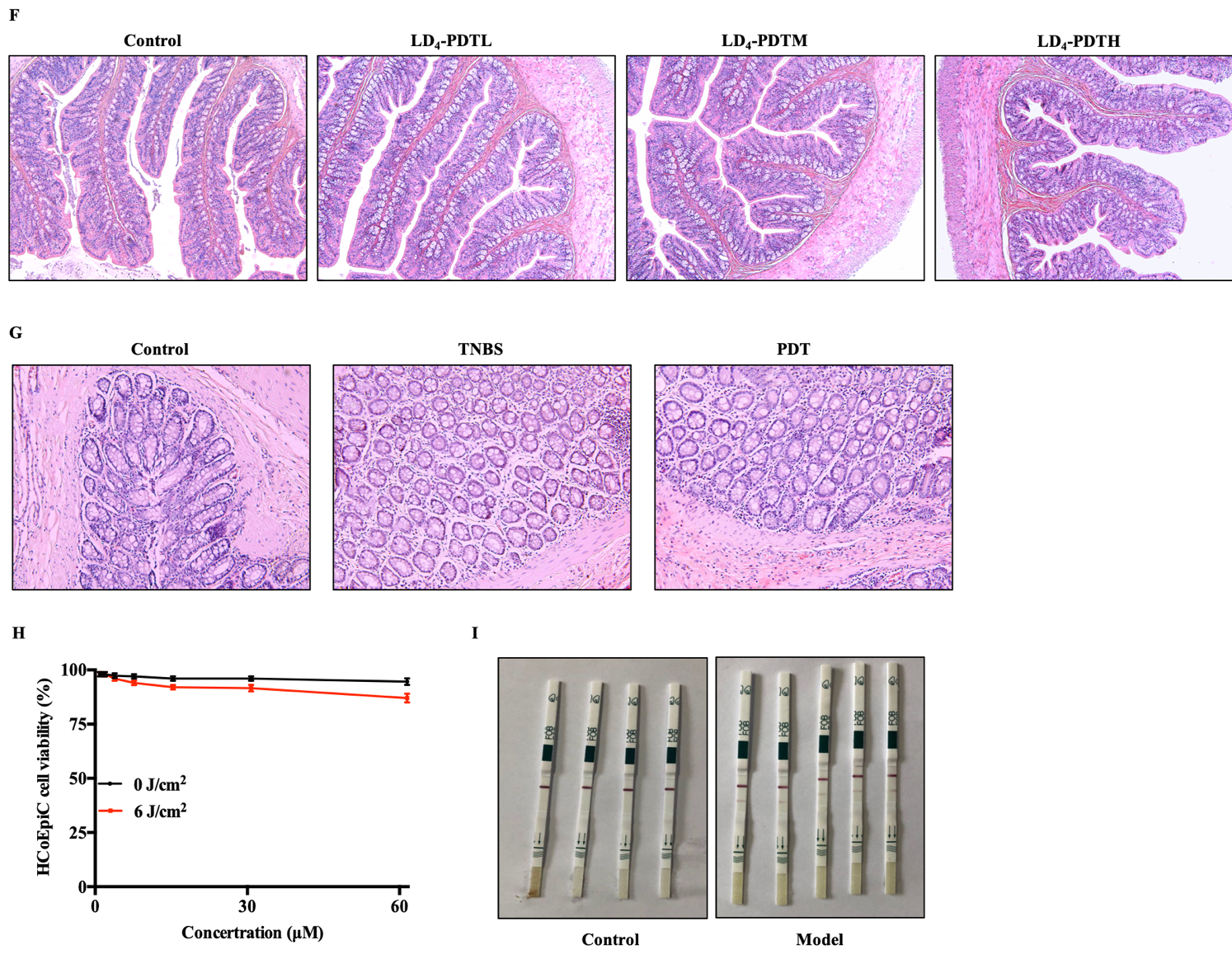
**

**Supplementary Figure S1.** (A) The chemical structure of LD_4_. (B) The schedule of experiments in SD rats. (C). The standard curve for FITC-dextran. (D) Typical standard curve for TNF-α, rat ELISA. (E). Typical standard curve for IL-6, rat ELISA. (F) Effects of three different doses of LD4 on normal rats (G) Effect of PDT alone on TNBS-induced colitis. (H) Cell viability of HCoEpiC cells incubated with LD_4_ alone (0–60 μM) was determined by MTT assay. Red line indicated the light reaction; black line indicated the dark reaction. Data are representative of three independent experiments, expressed as mean ± SD. (I) OB paper detected occult blood in stool sample.


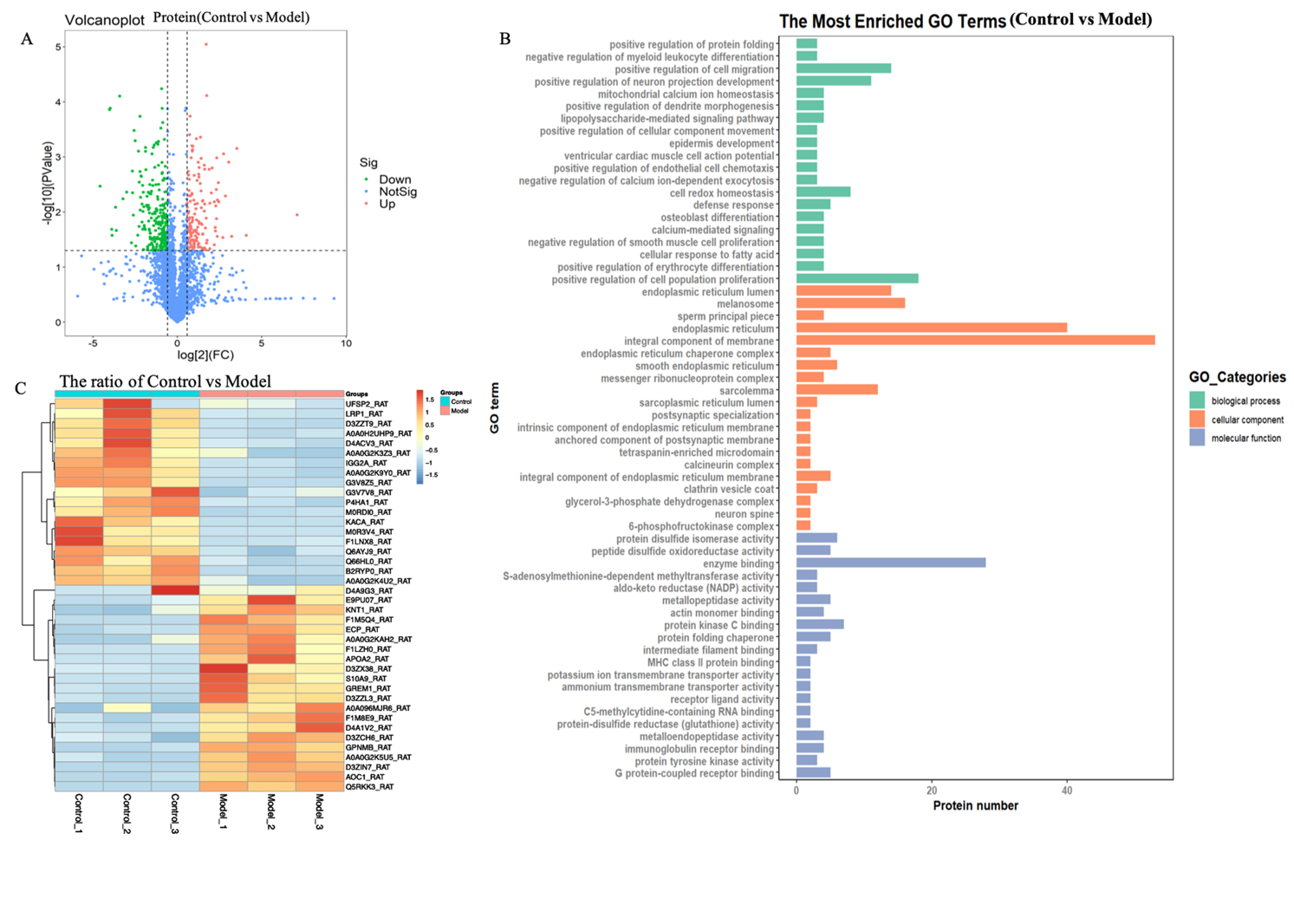


**Supplementary Figure S2.** LD_4_-PDT reprograms protein profile of colon tissues in TNBS-induced UC model rats

(A) Volcano plots showing the difference in protein expression between control and model groups. (B) All identified proteins were classified according to the first 20 GO ratios (sorted by enrichment degree), enrichment degree (-log10(*P-value*)) and gene number according to biological progress, cell composition and molecular function. Over-representation analysis was used for functional enrichment analysis, and the t test based on hypergeometric distribution. (C) Heat map of 40 differentially expressed proteins between control and model group. Each column represents a sample, and each row represents a factor. The red was the elevated protein and the green was the decreased protein.


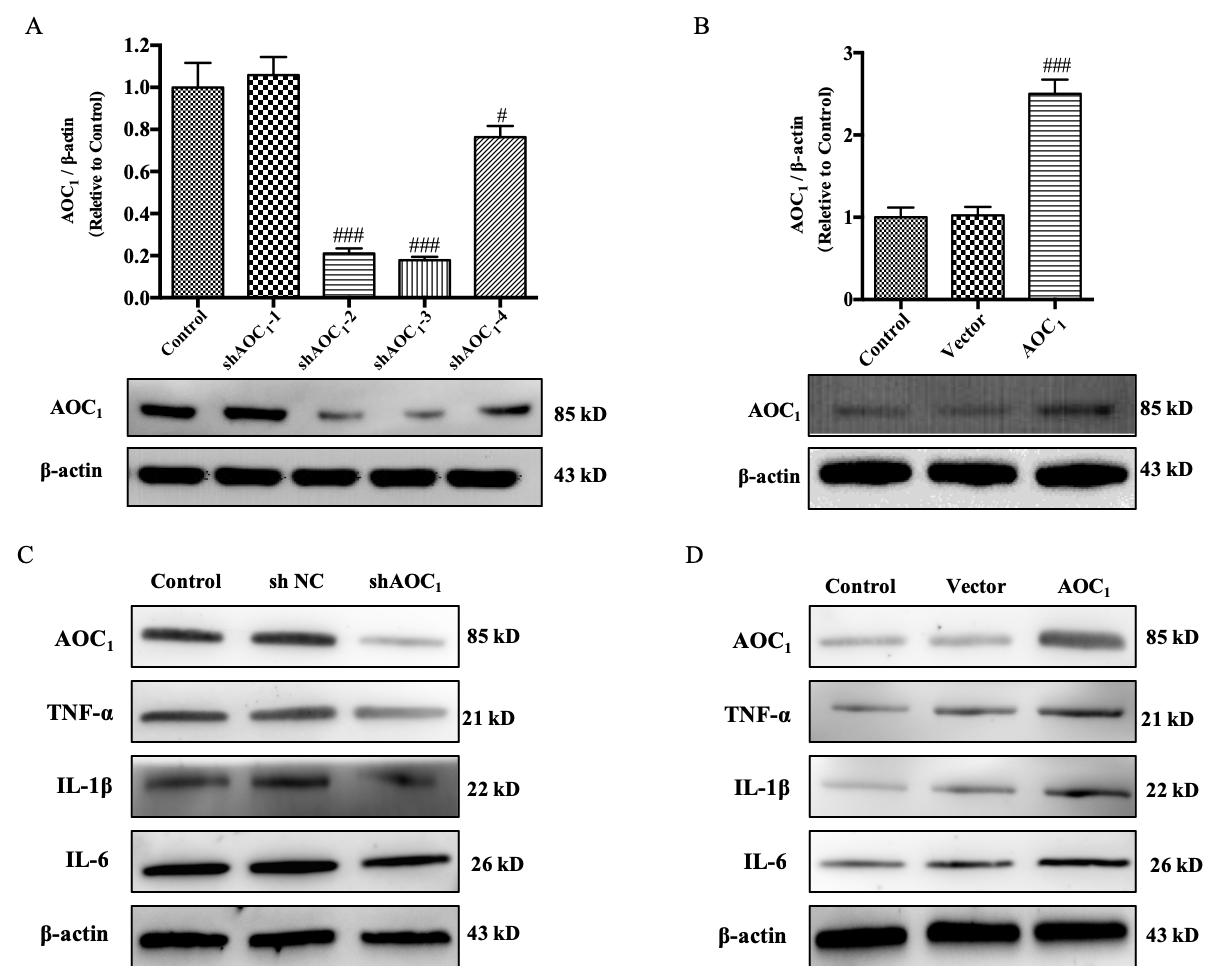


**Supplementary Figure S3.** The expression of AOC_1_

(A) Degree of AOC_1_ knockdown. (B) Degree of AOC_1_ over-expression. (C, D) Effects of overexpression or knockdown of AOC on *TNF-α,* *IL-6* and *IL-1* protein expression in HCoEpiC cells. ^#^*P<0.05,* ^##^*P<0.01*, ^###^*P<0.001* vs Control groups. Data are representative of three independent experiments, expressed as mean ± SD.


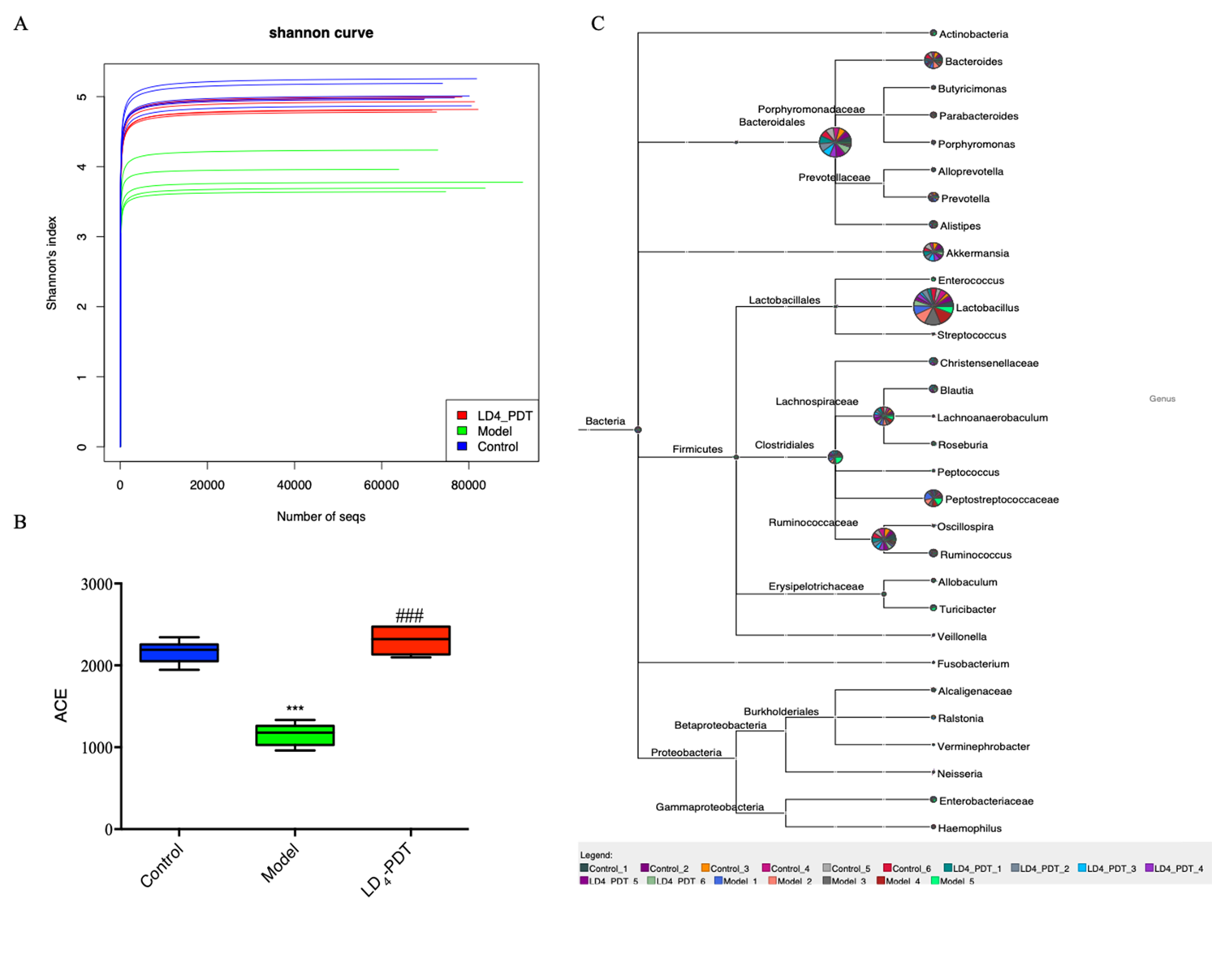


**Supplementary Figure S4.** LD_4_-PDT changes the gut microbiota in TNBS-induced UC model rats

(A) Shannon index α-diversity curve. The horizontal axis shows the number of sequences, and the vertical axis shows the diversity index. (B) The ACE index of intestinal bacteria in rats was examined by 16S rRNA high-throughput sequencing. (C) Final Megan simple tree.

**Supplementary Tables**

**Supplementary Table S1** Primers used for qRT-PCR.

| gene | Forward primer sequence 5'-3' | Reverse primer sequence 5'-3' |
| --- | --- | --- |
| AOC1 (homo) | TCATACAGCGCTATGTAGAAGG | CATAGAACTTCCCGTTGTACCA |
| AKT (homo) | TGACCATGAACGAGTTTGAGTA | GAGGATCTTCATGGCGTAGTAG |
| NF-κBp65 (homo) | TGTGAAGAAGCGGGACCTGGAG | AAGCAGAGCCGCACAGCATTC |
| TNF-α (homo) | CTCTTCAAGGGCCAAGGCTG | ATGGGCTCATACCAGGGCTT |
| IL-6 (homo) | CACTGGTCTTTTGGAGTTTGAG | GGACTTTTGTACTCATCTGCAC |
| IL-1 (homo) | GCCAGTGAAATGATGGCTTATT | AGGAGCACTTCATCTGTTTAGG |

**Supplementary Table S2** Sequences used for shRNA knockdown.

| gene | Sequence 5'-3' |
| --- | --- |
| Down-sh-AOC_1_-1 (homo) | GCTGAGGTTTCTGGATAAAGG |
| Down-sh-AOC_1_-2 (homo) | GCATCAGTTCTTCCTCCAATAC |
| Down-sh-AOC_1_-3 (homo) | GCCGCAGTTGGCTTATCATAC |
| Down-sh-AOC_1_-4 (homo) | GGATCATGGGAGCACAGATGC |

Data are available via ProteomeXchange with identifier PXD027815

**
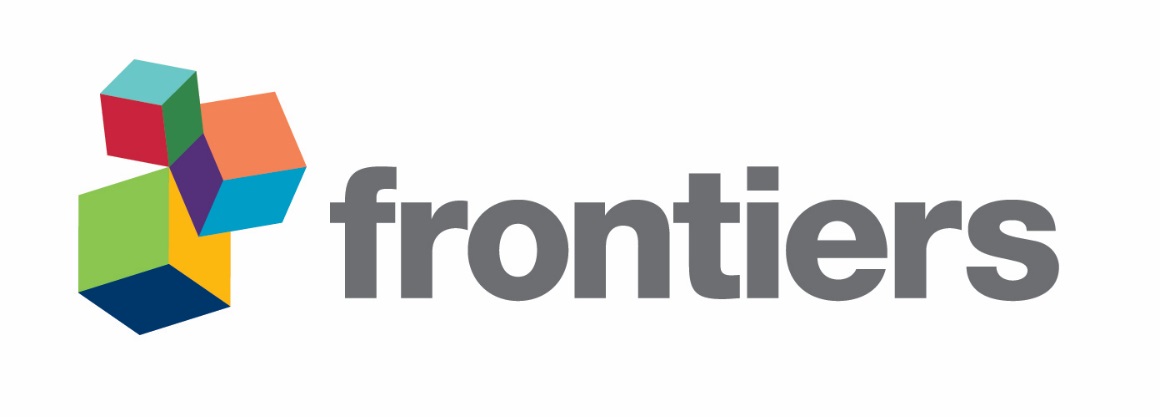
**
